# Effects of Carbohydrase Supplementation on Growth Performance, Intestinal Digestive Enzymes and Flora, Glucose Metabolism Enzymes, and *glut2* Gene Expression of Hybrid Grouper (*Epinephelus fuscoguttatus♀ × E. lanceolatus♂*) Fed Different CHO/L Ratio Diets

**DOI:** 10.3390/metabo13010098

**Published:** 2023-01-07

**Authors:** Hongyu Liu, Ling Pan, Jianfei Shen, Beiping Tan, Xiaohui Dong, Qihui Yang, Shuyan Chi, Shuang Zhang

**Affiliations:** 1 Laboratory of Aquatic Animal Nutrition and Feed, College of Fisheries, Guangdong Ocean University, Zhanjiang 524088, China; 2 Aquatic Animals Precision Nutrition and High-Efficiency Feed Engineering Research Center of Guangdong Province, Zhanjiang 524088, China; 3 Key Laboratory of Aquatic, Livestock and Poultry Feed Science and Technology in South China, Ministry of Agriculture, Zhanjiang 524088, China

**Keywords:** *Epinephelus coioides*, growth performance, carbohydrase, carbohydrate metabolism, *Glut2* gene

## Abstract

An optimal carbohydrate-to-lipid (CHO: L) ratio facilitates fish growth and protein conservation, and carbohydrase promotes nutrient absorption. Therefore, an 8-week feeding trial was conducted to investigate the effects of carbohydrase supplementation on growth performance, intestinal digestive enzymes and flora, glucose metabolism enzymes and *glut2* gene expression in juvenile hybrid grouper (*Epinephelus fuscoguttatus♀× Epinephelus lanceolatus♂*) fed different CHO: L ratios diets. L, M, and H represent CHO:L ratios of 0.91, 1.92 and 3.91, respectively. LE, ME, and HE represent CHO:L ratios of 0.91, 1.92, 3.91, respectively, supplemented with the same ratio of carbohydrase. Results showed that weight gain rate (WGR) and specific growth rate (SGR) reached a maximum in group M and were significantly enhanced by carbohydrase (*p* < 0.05). Crude lipid content decreased significantly with an increase in the dietary CHO:L ratio (*p* < 0.05). Significant increases in the trypsin (TRY) and amylase (AMS) activities and significant decreases in the lipase (LPS) activity were observed with increasing dietary CHO:L ratio, and the former two were significantly promoted by carbohydrase (*p* < 0.05). The content of liver and muscle glycogen increased significantly with the increasing dietary CHO:L ratio but decreased significantly after carbohydrase supplementation (*p* < 0.05). The glucokinase (GK), pyruvate kinase (PK), Phosphate 6 fructokinase-1 (PFK-1) and phosphoenolpyruvate kinase (PEPCK) activities increased significantly with increasing dietary CHO:L ratio (*p* < 0.05). *Glut2* mRNA expression decreased significantly in liver and increased significantly in intestine with increasing dietary CHO:L ratio (*p* < 0.05). By linear discriminant analysis (LDA), the abundance of *Alistipe* was significantly higher in Group ME than in Group M. These results suggested that hybrid grouper can only moderately utilize dietary carbohydrate and lipid in diet, and a certain amount of high glycemic lipids occurred when fed with high-carbohydrate diets. By the weight gain for basis, the supplementation of carbohydrase in Group H with amylase, glycosylase, and pullulanase in a 1:1:1 ratio effectively lowered glycemic lipids, promoted the growth of grouper, digestive enzymes activities and carbohydrate metabolic enzyme, and *glut2* gene expression in intestine, effectively balancing the negative effects of high-carbohydrate diet and improving the utilization of carbohydrate.

## 1. Introduction

Protein accounts for a significant portion of the cost of a formula diet. In addition to protein, carbohydrates and lipids are key nutrients in fish that have a significant function in the growth, development and immunity of fish [1,2]. Like other all animals, fish depend on energy from their foodstuff to fulfil their normal growth requirements. However, in contrast to other land animals, particularly when there is not enough energy in the diet, fish obtain their total energy requirements by the breakdown of proteins into amino acids [3]. With the development of intensive farming and the soaring price of fishmeal, research on the substitution and cost-effectiveness of protein in feed has been continuously conducted. A number of studies have demonstrated that an appropriate CHO:L ratio in feed can improve growth, feed efficiency, enzyme activity, protein conservation and reduce nitrogen and ammonia excretion, promoting an improved farming environment [4,5]. Most fish make good use of lipids, but at high dietary levels, they may lead to fatty liver in fish [6]; similarly, the amount of lipids added is also detrimental to the lipid deposition of fish, which may affect the organoleptic characteristics of the fillet, such as its flavor [7,8]. On the one hand, carnivorous fish have a lower carbohydrate utilization than omnivorous and herbivorous fish. On the other hand, carbohydrates are more readily available and cheaper relative to lipids, but excess addition of carbohydrates in feed may inhibit their growth and increase glycogen accumulation in the liver [9,10]. Therefore, maintaining an optimum carbohydrate and lipid (CHO/L) balance will be beneficial for fish growth performance, feed utilization, nutrient absorption, and deposition.

The secretion of certain specific digestive enzymes in fish is so low that many nutrients in plant materials cannot be easily digested [11]. A trend towards increased energy and nitrogen-free leachate digestibility was observed with exogenous enzyme supplementation. The addition of glycosidase complexes to rapeseed meal feeds also significantly increased feed digestibility and improve the growth performance of spot prawns *(Pandalus platyceros)* [12]. It is generally accepted that an important role in glucose-sensitive mechanisms is played by *glut2*, and that it is expressed in the sinusoidal membrane of hepatocytes, which execute bidirectional glucose transport in response to dietary and hormonal conditions [13]. It has been previously shown that *glut2* expression in the liver of Atlantic cod (*Gadus morhua*) diminished during starvation and was boosted on refeeding; thus, *glut2* is thought to reflect the transport of glucose once the glycogen has been depleted [14]. At physiological conditions, the glucose transport rate of *glut2* varies with glucose concentration even under diabetic conditions [15]. Earlier studies reported that glycosylase supplementation in a high-carbohydrate diet significantly promoted the expression of *glut2* gene in liver and intestine [16]. From the current research situation, different CHO:L ratios have been studied in many species of fish [1,2], but there are few studies on how to mitigate the adverse effects on high CHO:L ratio. In our research, we investigated the effects on *glut2* gene expression at different CHO:L ratios and the supplementation of carbohydrase at different CHO:L ratios.

Hybrid grouper (*Epinephelus fuscoguttatus♀* × *E. lanceolatus♂*) possesses the advantages of excellent growth, high illness resistance and commercial value, and represents a typical representative of a carnivorous fish. More recently, it has emerged as the dominant species of grouper farmed in southeast China [17]. In order to maximize profits, diets are often formulated to contain high levels of CHO:L ratio to reduce feed costs. Although the optimal glycolipid ratio requirements at the juvenile stage of grouper have been studied [18,19,20], there is little research on the addition of carbohydrase based on different glycolipid ratios. With this in mind, the purpose of this study was to assess the influence of carbohydrase supplementation on growth performance, carbohydrate metabolic enzyme and *glut2* gene expression in the Hybrid Grouper-fed diets with different CHO:L ratios.

## 2. Materials and Methods

### 2.1. Experimental Diets and Animals

The experimental formula was based on fish meal and casein as protein source, fish oil and phospholipid oil as fat source, and corn starch as sugar source, in which the protein level was about 43%. Lysine and methionine were added to ensure amino acid growth balance for juvenile [21]. The carbohydras was provided by Guangdong VTR Biotechnology Co., Ltd (Zhuhai, China). and added in the recommended amount. Six iso-nitrogenous and iso-energetic diets were provided, respectively. L, M, and H represent CHO:L ratios of 0.91, 1.92 and 3.91, respectively. LE, ME, and HE represent CHO:L ratios of 0.91, 1.92, and 3.91, respectively, supplemented with the same ratio of carbohydrase. Amylase (enzyme activity of 10,000 U/g product), glycosylase (enzyme activity of 100,000 U/g product), and pullulanase (enzyme activity of 21,000 U/g product) are components of carbohydrase and are added in a ratio of 1:1:1. After crushing and passing through a 60 mesh screen, the ingredients were accurately weighed and evenly mixed to make a hard pellet feed about 2.5 mm in diameter. Formulation and proximate composition of the experimental diets are presented in Table 1. Pelleting was performed using a twin-screw extrude (South China University of Technology, Guangzhou, China), and all feeds were naturally air-dried at room temperature and then stored at −20 °C in plastic Ziploc bags until used. 

The hybrid groupers used in the experiments were purchased from the Hongyun Fish Hatchery (Zhanjiang, China). They were raised at the Donghai Island Marine Life Base of Guangdong Ocean University for 2 weeks. Before the feeding test, it adapted to the experimental conditions by feeding on commercialized food. Then, healthy fish with no obvious weight difference (average weight: 9.55.0 ± 0.5 g) were chosen and were randomly divided into three repeat groups, with 30 fish in every tank (0.5 m^3^), for a full meal each day (8:30–16:30). The feeding test was carried out for 8 weeks, during which the water temperature was between 26 and 30 °C and the pH was between 7.2 and 7.4. The dissolved oxygen and total ammonia nitrogen remained above 5.0 mg/L and below 0.2 mg/L, respectively. 

### 2.2. Sample Collection and Chemical Analysis

At the end of the 8-week feeding trial, the fish were starved for 24 h before samples were collected. All fish in each tank were collected and weighed for analysis of weight gain rate (WGR), specific growth rate (SGR), feed conversion ratio (FCR), and survival rate (SR). After weighting and counting, five fish from each tank were randomly selected to collect blood using a sterile 1 mL syringe in 1.5 mL Eppendorf tubes with an ice-water bath, and finally stored at 4 °C for 6 h [22] before centrifugation. The supernatant was collected for analysis of plasma biochemical parameters. Three fish from each tank were collected and stored at −20 °C for analysis of whole-body composition. Livers and intestines from three fish were quickly removed, frozen in liquid nitrogen and stored at −80 °C to analyze the glycolytic enzyme activity of the liver and the digestive enzyme activity of the intestine. The liver and muscle of three fish were collected from each tank to determine glycogen content with alkaline digestion kit bought from Nanjing Jiancheng Institute of Bioengineering Engineering. This was performed in strict accordance with the kit instructions for all test reagent preparation, sample preparation and precautions. Liver and intestine samples of the other two fish were immersed in 1 mL RNA later RNA stabilization reagent (Qiagen, Valencia, CA, USA), frozen in liquid nitrogen and stored at −80 ℃ until RNA extraction.

### 2.3. Proximate Composition Analysis

Diets and fish were analyzed for proximate composition. Moisture was determined by oven drying at 105 °C until constant and ash by combustion at 550 °C for 4 h and crude ash by fritted glass crucible method using an automatic analyzer (ANKOM A2000i, Macedon, New York, NY, USA). Crude protein (Nitrogen 9 6.25) was determined by Kjeldahl method using an automated Kjeldahl system (FOSS KT260, Hillerød, Denmark). Crude lipid was obtained by ether extraction using an Auto Soxtec System (Soxtec HT6 Tecator, Haganas, Sweden). Total energy was determined by 5E-1C automatic calorimeter Kaiyuan apparatus in diets. The standard procedure (Association of Official Analytical chemists, AOAC, 2005) was used to carry out approximate analyses to determine the moisture content, crude protein and crude lipid in diets.

### 2.4. Plasma Biochemical Indexes and Glycogen Content Assay

Blood glucose (GLU, F006-1-1), plasma insulin (INS, H203-1-2), total cholesterol (CHOL, A111-1-1), and triglycerides (TG, A110-1-1) were determined by commercial kits. Liver and muscle glycogen were measured by spectrophotometer at 620 nm with a glycogen kit. All kits were obtained from Nanjing Jiancheng Institute of Biological Engineering, China. All test reagent preparation, precautions samples and preparation were carried out strictly according to the instructions of the kit.

### 2.5. Digestive Enzyme and Glucose Metabolic Enzyme Activity Assay

In short, we weighed the intestinal samples according to the previously described method [16], added to 9 times the volume of saline in proportion to the weight and volume of 1:9, mechanical homogenization in the ice bath and collected the liquid in a 1.5 mL Eppendorf tube and centrifuged at 2500× *g* for 20 min at 4 °C to analyze the activity of the digestive enzymes. The activities of trypsin (TRP, JL22682), lipase (LPS, 1L22695) and amylase (AMS, JL37039) were analyzed by colorimetry using purchased commercial kits, followed by spectrophotometry at 253, 540 and 660 nm wavelengths, respectively. Readings were taken from the photometer, and the company’s instructions were strictly followed. After slicing and weighing the liver, PBS was added (PH 7.2–7.4), and it was frozen quickly in liquid nitrogen; the samples were maintained at 2–8 °C after melting, PBS was added (PH 7.2–7.4), and it was homogenized by hand or grinders and centrifuged at 2000–3000 rpm for 20 min to retain the supernatant. The activities of GK, PK, PFK-1 and PEPCK were determined by colorimetric analysis, using purchased commercial kits, then read at a wavelength of 450 nm, and carried out in strict accordance with the company’s instructions. The activity of the enzyme is expressed as the concentration of total protein per milligram, which is determined by spectrophotometry [5,23]. All the above kits were provided by Shanghai Enzyme-linked Biotechnology Co., Ltd., (Shanghai, China). The preparation of all test reagents, preventive samples and shores were carried out in strict accordance with the instructions of the kit.

### 2.6. Real-Time Quantitative RT-PCR Analysis of Gene Expression

The reaction expression of *glut2* in response to different CHO:L ratios and carbohydrase in the diet was studied by RT-PCR. The liver and muscle of two fish per tank were kept for total RNA extraction, and the total RNA was extracted with a Trans Gen Biotech (Beijing, China) RNA kit. The extracted RNA was checked for RNA quality and concentration by agarose gel electrophoresis and spectrophotometry. The extracted RNA was reverse-transcribed to cDNA using Accurate Biology Evo M-MLV kit (Hunan, China), and finally, the obtained cDNA was stored in a refrigerator at −20 °C for real-time quantification. Specific primers for RT-PCR were designed according to the published sequence of groupers (Table 2), and β-Actin was used as house-keeping gene after its stability was verified [24]. All real-time PCR reactions with volumes of 10 µL (1 µL cDNA, 0.8 µL primer, 3.2 µL RNseFree dH_2_O, and 5 µL SYBR^®^ Green Real-Time PCR Master Mix) were conducted on a Light Cycler 480 Biosystems Real-Time PCR system (Shanghai, China). Finally, the data were copied from the machine, and we used β-actin as the reference gene to calculate the relative gene expression by the 2^−ΔΔCT^ method [25].

### 2.7. Intestine Microbiological Analysis

The V4 region of the intestinal 16S r DNA gene was sequenced by the Illumina Mise q method developed by Beijing Novo zyme Bioinformatics Co. The V4 region of the intestinal 16S r DNA gene was sequenced by Beijing Novo Horizon Bioinformatics Co. After sequencing, the two Illumina short reads were compiled according to the overlap, and the fast q file was processed to generate the fast q file. The fast q files were compiled and processed to generate the respective fast a and qual files, and then statistically analyzed using standard methods. From the V4 region, 16S r NDA sequences were extracted from six samples, and the resulting sequences were classified into different OTUs. The obtained sequences were classified into species using RDP classifier software.

### 2.8. Statistical Analysis

Before applying ANOVA, normal and homogeneity analysis were carried out. In this article, two analytical methods were used in total, and all data were presented as mean ± SD (n = 3). First of all, the Tukey’s post hoc multiple range test in one-way ANOVA analysis of all data was carried out to test the differences between all diets. Finally, two-way ANOVA and Tukey’s multiple range test were used to analyze the significance of the mean between treatments with respect to the interaction between multiple dependent variables. The analysis of all data was operated through SPSS software 19 (Chicago, Michigan Avenue, IL, USA).

## 3. Results

### 3.1. Growth Performance, Survival and Feed Utilization

Growth performance, survival rate and feed utilization of groupers fed the experimental diets are shown in Table 3. The weight gain (WGR), specific growth rate (SGR), and feed conversion ratio (FCR) of groupers were significantly (*p* < 0.05) affected by the CHO:L ratios and carbohydrase factors, but the impact of interaction was not significant (*p* > 0.05). Carbohydrase supplementation a high carbohydrate diet displayed the best growth performance. Significant improvements in FBW, WGR, SGR were observed after carbohydrase supplementation in different CHO:L ratios (*p* < 0.05). FCR was significantly affected by different CHO:L ratios (*p* < 0.05), but not significantly affected by carbohydrase (*p* > 0.05). There was no significant effect on SR between the treatment groups (*p* < 0.05).

### 3.2. Whole-Body Composition Analysis

The effects of carbohydrase supplementation on the conventional nutrient composition of hybrid grouper fed the experimental diets are shown in Table 4. Whole-body moisture, crude protein and ash were not significantly influenced by dietary CHO:L ratios and carbohydrase (*p* > 0.05). However, whole-body lipid was significantly affected by dietary CHO:L ratio (*p* < 0.05), but the presence or absence of carbohydrase had no significant impact on whole-body crude lipid content (*p* > 0.05). The interaction between different CHO:L ratios and carbohydrase in the diet had no effect on whole-body composition (*p* > 0.05).

### 3.3. Plasma Biochemical Composition Measurement

The effects of carbohydrase supplementation on plasma biochemistry in the hybrid grouper fed the experimental diets are shown in Table 5. The plasma biochemical parameters were significantly influenced by the CHO:L ratio and carbohydrase factors (*p* < 0.05). The plasma TG and INS concentrations were significantly affected by dietary CHO:L level and carbohydrase (*p* < 0.05), but there was no significant interaction between either (*p* > 0.05). The plasma TG and INS concentration reached, respectively, minimum and maximum values in group H with dietary different CHO:L ratios, and a significant effect was present with and without carbohydrase (*p* < 0.05). Plasma TC and GLU levels were significantly impacted by dietary CHO:L ratios and carbohydrase (*p* < 0.05), and the interaction effect of both was significant (*p* < 0.05). A significant decrease in plasma TC and GLU level was observed after carbohydrase supplementation on different CHO:L ratios, particularly in Group H (*p* < 0.05).

### 3.4. Intestinal Digestive Enzyme Activities Analysis

Effects of carbohydrase supplementation on intestinal digestive enzymes in hybrid grouper fed different CHO:L ratios diets were shown in Table 6. As can be seen in the table, the intestinal digestive enzyme activities of groupers were significantly influenced by the CHO/L ratio and carbohydrase factors (*p* < 0.05). The trypsin activity was significantly affected by dietary CHO:L ratio and carbohydrase (*p* < 0.05) and reached a maximum value in Group H, but the impact of interaction was not significant (*p* > 0.05); the lipase activity was significantly influenced by dietary CHO:L level (*p* < 0.05) and reached a minimum value in Group H, but carbohydrase did not have a significant effect (*p* > 0.05); the amylase activity was significantly influenced by dietary CHO:L level and carbohydrase (*p* < 0.05), and there was a significant interaction (*p* > 0.05), which reached a maximum value in Group HE. 

### 3.5. Glycogen Content Assay

**Figure 1:**
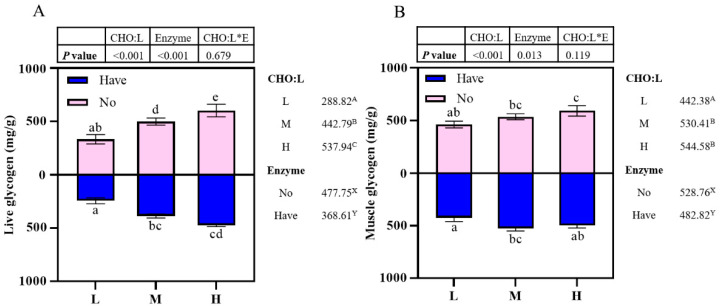
Effects of carbohydrase supplementation on glycogen content in hybrid grouper fed different CHO:L ratios diets (Figure 1A = Liver glycogen; Figure 1B = Muscle glycogen). Data represent means of three fish in each group; error bar indicates S. D. Average of different superscripts in the same column (a, b, c, d, e, etc. Or A, B, C, etc. Or X, Y) are significantly different (*p* < 0.05). (Dietary CHO:L ratio = A, B, C, etc.; Dietary carbohydrase = X, Y); no means no enzyme, have means enzyme.

Effects of carbohydrase supplementation on glycogen content in hybrid grouper fed different CHO:L ratios diets are shown in Figure 1. Liver glycogen content was significantly affected by dietary CHO:L level and carbohydrase (*p* < 0.05), which accumulated with an increased dietary CHO:L level, which decreased significantly after carbohydrase supplementation with dietary different CHO:L ratios (*p* < 0.05), but there was no significant interaction between the two dependent variables (*p* > 0.05) (Figure 1A). Muscle glycogen content was affected by dietary CHO:L level and carbohydrase (*p* < 0.05), which increased slowly with an increase in dietary CHO:L level, reaching a maximum in Group H and decreasing significantly after supplementation (*p* > 0.05).

### 3.6. Enzyme Activity Related to Carbohydrate Metabolism in the Liver

**Figure 2:**
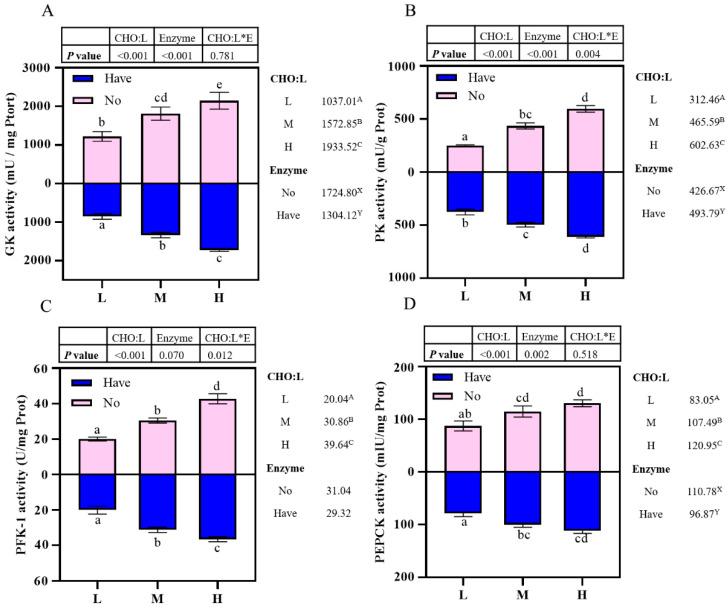
Effects of carbohydrase supplementation on hepatic enzyme activities in hybrid grouper fed different CHO:L ratios diets (Figure 2A = GK activity; Figure 2B = PK activity; Figure 2C = PFK-1 activity; Figure 2D = PEPCK activity). Data represent means of three fish in each group; error bar indicates S. D. Average of different superscripts in the same column (a, b, c, d, e, etc. Or A, B, C, etc. Or X, Y) are significantly different (*p* < 0.05). (Dietary CHO:L ratio = A, B, C, etc.; Dietary carbohydrase = X, Y); no means no enzyme, have means enzyme.

Effects of carbohydrase supplementation on hepatic gluconeogenic enzyme activities in hybrid grouper fed different CHO:L ratios diets are shown in Figure 2. Hepatic enzymes activities were significantly affected by dietary CHO:L ratio and carbohydrase index (*p* < 0.05). The GK and PEPCK activities were significantly influenced by dietary CHO:L level and carbohydrase (*p* < 0.05), which accumulated with an increase dietary CHO:L level and decreased significantly after carbohydrase supplementation (*p* < 0.05), reaching a maximum value in Group H, but no significant interaction was observed (*p* > 0.05) (Figure 2A,D). The PK activity was significantly affected not only by dietary CHO:L ratio, but also by carbohydrase (*p* < 0.05), and had an interactive effect between the two variables (Figure 2B). Dietary CHO:L ratio significantly promoted the PFK-1 activity (*p* < 0.05), with the highest value in Group H. In addition, the PFK-1 activity was significantly affected by the interaction between dietary CHO:L level and carbohydrase (*p* < 0.05), but dietary carbohydrase did not have a significant effect (*p* > 0.05) (Figure 2C).

### 3.7. The Impact of Different Diets on glut2 mRNA Gene Expression

**Figure 3:**
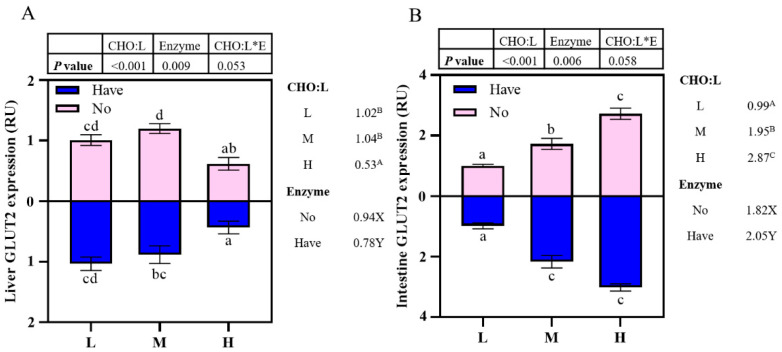
Effects of carbohydrase supplementation on *glut2* mRNA expression in hybrid grouper fed different CHO:L ratios diets (Figure 3A = Liver *glut2* expression; Figure 3B = Intestine *glut2* expression). Data represent means of three fish in each group; error bar indicates S. D. Average of different superscripts in the same column (a, b, c, d, etc. Or A, B, C, etc. Or X, Y) are significantly different (*p* < 0.05). (Dietary CHO:L ratio = A, B, C, etc.; Dietary carbohydrase = X, Y); no means no enzyme, have means enzyme.

The effects of carbohydrase supplementation on *glut2* mRNA expression in hybrid grouper fed different CHO:L ratio diets are shown in Figure 3. The relative expression of *glut2* mRNA in the liver decreased significantly with an increase in dietary CHO:L ratio (*p* < 0.05), reaching the lowest value in Group H. In addition, the presence or absence of carbohydrase caused significant effects on hepatic *glut2* expression (Figure 3A). High dietary CHO:L level significantly promoted *glut2* expression in the intestine (*p* < 0.05), and carbohydrase resulted in significant differences (*p* < 0.05). Interactions between dietary CHO:L levels and carbohydrase were absent in both liver and intestinal *glut2* expression (*p* > 0.05).

### 3.8. The Impact of Different Diets on Intestinal Microflora

#### 3.8.1. Analysis at the Phylum and Genus Level

**Figure 4:**
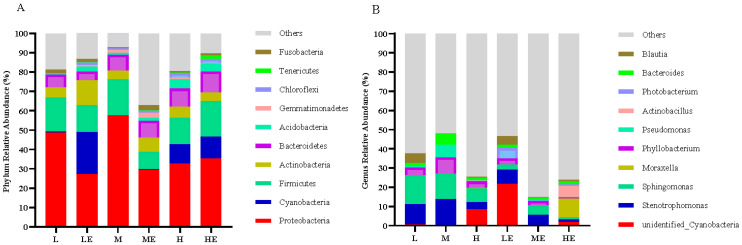
Intestinal microbial composition in grouper (Figure 4A = Phylum relative abundance; Figure 4B = Genus relative abundance).

Effects of carbohydrase supplementation on intestinal microflora in hybrid grouper fed different CHO:L ratios diets were shown in Figure 4. Among the top ten horizontal phylum, the phylum *Proteobacteria*, *Cyanobacteria*, *Firmicutes*, *Actinobacteria*, *Bacteroides*, *Acidobacteria*, *Gemmatimonadetes*, *Chloroflexi*, *Tenericutes*, *Fusobacteria* are relatively abundant and dominant (Figure 4A). Among the top ten genera, *Cyanobacteria*, *Stenotrophomonas*, *Sphingomonas*, *Moraxella*, *Phyllobacterium*, *Pseudomonas*, *Actinobacillus*, *Photobacterium*, *Bacteroides*, *Blautia* are relatively abundant and dominant (Figure 4B).

#### 3.8.2. Multi Sample Comparative Analysis

**Figure 5:**
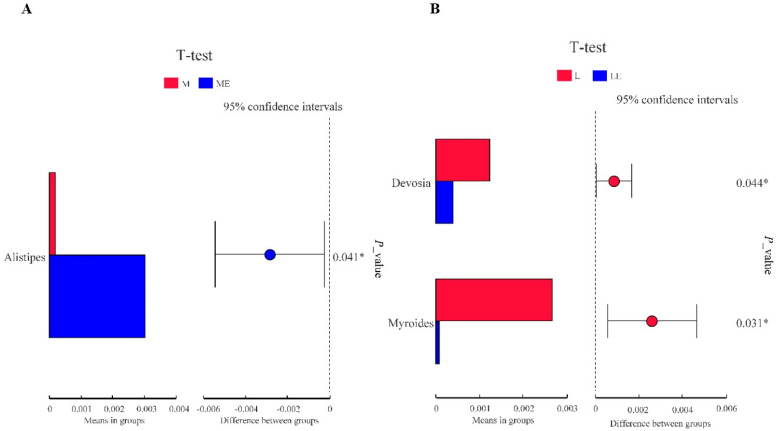
Importance *t*-test for functional variance analysis (Figure 5A= groups M and ME by *t*-test; Figure 5B = groups L and LE by *t*-test). “*” indicated significant difference at the *p* < 0.05 level.

We used the T-test to analyze the differences in intestinal floral of grouper taxa between the group without and with enzymes (Figure 5). As shown in Figure 5A, the relative abundance of *Alistipes* in Group ME was significantly higher than that in the M group; as shown in Figure 5B, the relative abundance of *Devosia* and *Myroides* in Group LE was significantly lower than that in the L group (*p* < 0.05). 

#### 3.8.3. Linear Discriminant Analysis

**Figure 6:**
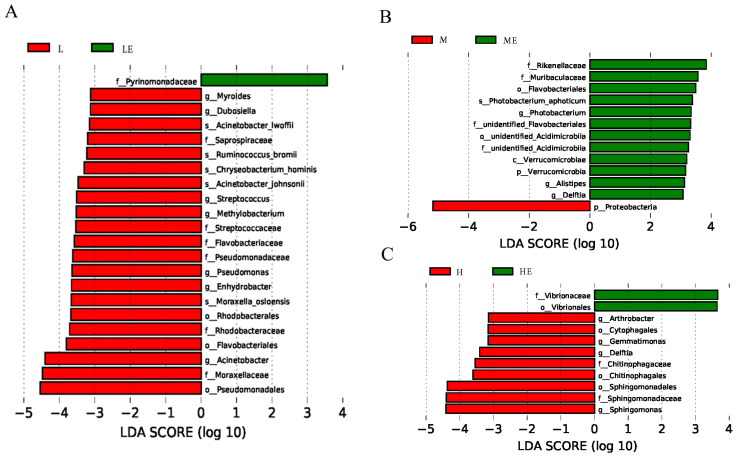
Linear discriminant analysis effect size (Lef Se) results revealed differences in intestinal flora between enzyme-free and enzyme groups (Figure 6A = groups L and LE by Lef Se; Figure 6B = groups M and ME by Lef Se; Figure 6C = groups H and HE by Lef Se). LDA score > 3, *p* < 0.05.

We used linear discriminant analysis (LDA) to analyze the differences in intestinal floral of grouper taxa between the group without and with enzymes (Figure 6). The results of Lef Se analysis showed that the abundance of *Pyrinomonadaceae* were significantly higher in Group LE than in Group L (*p* < 0.05, Figure 6A); the abundance of *Rikenellaceae*, *Muribaculaceae*, *Flavobacteriales*, *Photobacterium*, *Acidimicrobiia*, *Verrucomicrobiae*, and *Alistipe* was significantly higher in Group ME than in Group M (*p* < 0.05, Figure 6B); the abundance of *Vibrionaceae* and *Vibrionales* was significantly higher in Group HE than in Group H (*p* < 0.05, Figure 6C).

## 4. Discussion

Carbohydrates are considered to provide energy at a low cost, while lipids are essential for animal growth and development, and the provision of an appropriate amount of CHO: L ratios in the diet can have beneficial effect on animal growth and spare the utilization of fishmeal protein [2,26,27,28]. With the scarcity of fishmeal resources, the impact of different levels of dietary CHO: L ratios on growth have been assessed in a number of aquaculture species with varying results, such as grass carp (*Ctenopharyngodon idellus*) [5], Nile tilapia (*Oreochromis niloticus*) [2], juvenile yellow catfish (*Pelteobagrus fulvidraco*) [26], and juvenile Senegalese sole (*Solea senegalensis, Kaup*) [4]. In recent years, exogenous enzymes have been extensively studied in livestock and poultry as functional additives to improve the nutritional digestibility of plant-based raw materials. Therefore, in the present study, we not only investigated the impact of CHO: L ratios, but also explored the impact of carbohydrase. Our results showed that fish fed CHO:L ratio of 1.92 achieved the best growth performance compared to fish fed CHO:L ratios of 0.91 and 3.91. Previous studies on groupers [18,29] have also shown that the best growth was achieved at medium CHO:L ratio, which was similar to our results. A significant increase in WG and SGR and a significant decrease in FCR were observed with carbohydrase supplementation at a CHO:L ratio of 3.91, indicating that carbohydrase act as a growth promoter. In studies on Atlantic salmon (*Salmo salar*) [30], tilapia [31], and large yellow croaker (*Larimichthys crocea*) [28], it was found that appropriate CHO: L ratios improved growth performance and feed digestibility, but higher CHO: L ratio conditions were found to limit carbohydrate absorption in various fish species. The ability of carnivorous fish to utilize carbohydrates in their diet was known to be relatively low compared with grass carp and tilapia, and also to lead to different effects. Hybrid grouper was able to tolerate 28% of dietary starch with no negative impact on growth performance [32]. The experimental group with a CHO:L ratio of 3.91 contained 33% starch, which may be responsible for the poorer growth of the groupers. A report on rohu (*Labeo rohita*) showed a significant improvement in growth and protein utilization after 50 mg/kg α-amylase supplementation in non-gelatinized corn diet [33]. Amylase is known to hydrolyze large α-linked polysaccharides (such as starch, glycogen, branched-chain starch), while glycosylase and pullulanase are exogenous enzymes that can hydrolyze the glycoside bonds of type α-1,4 and α-1,6, releasing D-glucose by continuously consuming the glucose unit at the non-reduction end of the sugar chain [34]. Protein-sparing effects and protein-carbohydrate hydrolysis may occur when exogenous enzymes supplemented to the diets [35]. This may account for better growth performance of grouper-fed carbohydrase supplementation at a CHO:L ratio of 3.91.

There were no significant differences in moisture, ash, and crude protein content between all treatment groups. In contrast, crude lipid decreased significantly with the increase in dietary CHO:L ratio, which was consistent with previous reported on large yellow croaker [28], hybrid Clarias catfish (*Clarias macrocephalus × C. gariepinus*) [36], and juvenile hybrid grouper [19]. When nutritional requirements are fulfilled, the appropriate lipid content in the diet is about 10% for hybrid grouper [37]. Higher lipid content in the diet, which cannot be fully utilized by grouper and tended to cause lipid deposition in different tissues, whereas the amount of lipid content obtained from carbohydrate was probably quite limited. This result confirms a positive correlation between lipid deposition in fish and the lipid content of the diet [28,38,39]. As we all know, the levels of triglycerides, cholesterol, glucose and total protein in blood are generally correlated with animal health and indicate the metabolic and physiological state of the organism, and too high or too low in blood will cause aberrant changes in the body [40]. It is well-known that changes in tissue cholesterol concentrations depend on the nutritional status of the fish [41]. Among the plasma biochemical indicators of our research, a significant decrease in TG and TC concentration was observed with the decrease in dietary lipid level, which suggested that endogenous lipid transport is more dynamic in response to higher lipid in the diet. This indicates that lipid transport in the blood is quite efficient in the presence of high lipids, and the results of this study are in consistency with earlier research reported on juvenile yellow catfish [2,5]. Contrastingly, plasma insulin and glucose increased with the increase in dietary CHO:L ratio. Long-term adaptation to high carbohydrates can increase plasma insulin concentrations in fish [42]. In mammals, promoting glucose uptake by the liver and skeletal muscle, increasing glucose uptake and fatty acid influx are the most prominent functions of insulin [43,44]. In fact, most dietary carbohydrate is then excreted after digestion, either stored as glycogen in the body or converted to fat [28]. Insulin levels were highest in fish fed a CHO:L ratio of 3.91, and we also found that glycogen content both in liver and muscle increased significantly with the increase in dietary CHO: L ratios, which was a reflection of the self-balancing mechanism of internal environmental homeostasis in fish. 

Fish use glucose in a way similar to that of mammals, alternating glycolysis and glycogenesis to maintain glucose balance [45]. Many studies have found that carbohydrate in the diet can regulate the activity of GK and its gene expression [46,47,48,49]. The primary function of GK, one of the four hexokinases, is to remove glucose from the blood after meals. Some studies have reported that the GK activity in the liver of European sea bass *(Dicentrarchus labrax)* and gilthead seabream (*Sparus aurata*) increased significantly with dietary starch level from 10% to 20% [50,51,52]; however, as the starch level increased from 20% to 30%, GK activity in the liver stopped increasing, indicating it may be close to threshold for the ability to utilize glucose effectively [52]. In this study, GK activity increased significantly with increasing carbohydrate in the diet, which was similar to the studies described above. In contrast, GK activity was significantly reduced by the addition of complex enzymes to the feed that enhanced the hydrolysis of starch, whereas small-molecule carbohydrates are better utilized by fish than large-molecule carbohydrates [53], which may be responsible for the reduction in GK activity. Besides GK, PK and PFK-1 are also important rate-limiting enzymes in glycolysis. In this study, PFK-1 activity increased significantly with the increase in dietary CHO: L ratios, and there was no significant difference with the addition of carbohydrase. As in mammals, PFK-1 activity in the liver of fish fed a high-carbohydrate, low-protein diet was higher than in those fed a high-protein, low-carbohydrate diet [54,55]; PK activity elevated significantly with the increase in dietary CHO: L ratios, and also with carbohydrase supplementation. However, previous studies have shown that the supplementation of 30% starch or glucose in the diet of gibel carp (*Carassius auratus gibelio*) has no significant effect on PK activity of livers [56]. This is due to differences in the regulation of PK activity by the content and source of carbohydrate in the feed. In our research, PEPCK activity followed a similar trend to GK activity and was regulated by CHO: L ratios and carbohydrase. However, the ingestion of digestible sugars can lead to a decrease in PEPCK activity and gene expression reported in common carp (*Cyprinus carpio*) [57]. In contrast, the PEPCK activity and gene expression of Atlantic salmon and rainbow trout (*Salmo gairdneri*) were not influenced by the digestible sugar content of the diet [52,58,59]. Therefore, further research is needed to explore this aspect.

Glucose enters the circulatory system in the digestive tract mainly through the transport of glucose transport carriers across the cell membrane [60]. The transport of glucose into the cell is the first step in the utilization of glucose by all tissues. Molecular evidence suggested that the main function of glucose transporter 2 (*glut2*) in fish is to transport high concentrations of glucose from the intestine to the blood and from the blood to hepatocytes [61,62]. As with mammals, *glut2* was as well expressed in the liver and intestine of sea bass [63] and rainbow trout [64]. In the present study, *glut2* expression in the liver was significantly decreased with an increase in the dietary CHO:L ratio, while it was upregulated in the intestine. Earlier studies have shown that glucoamylase supplementation on a high-carbohydrate diet significantly promoted *glut2* gene expression in the intestine of pompano (*Trachinotus ovatus*) [16]. Higher glucose concentrations in the intestine have an up-regulatory effect on *glut2* expression. It was shown that the intake of glucose increased with the increase in dietary carbohydrate, and then glucose was rapidly transported by *glut2* in the intestine to maintain glucose homeostasis and sustained energy supply. In our experiments, enzyme supplementation did not cause significant effects, but there was a slight upward trend, which could be related to the different carbohydrate sources used and the different carbohydrase.

Ultimately, digestive and absorptive capacity plays a crucial role in the growth and development of fish [65], and endogenous metabolic enzymes are one of the factors that determines the ability of fish to use dietary carbohydrates as an energy source [66]. In this experiment, intestinal amylase and trypsin increased with the increase in dietary CHO: L ratio, while lipase activity decreased. The results of this study were in agreement with previous research reported on groupers [19], in juvenile cobia (*Rachycentron canadum*) [67]. Reference [67] showed that the activity of amylase and protease significantly increased with the increase in starch level from 1.3% to 12.5%. Reference [20] suggested that the activity of amylase and trypsin in the intestine increased significantly with the varying of CHO: L ratios from 0.65 to 8.51, and a decreasing trend in lipase. The reason for this difference was that even for the same fish, the CHO: L ratio was smaller and the lipid content was lower in our study, and the digestive enzyme activity in the intestine was induced by the food level, which had a significant difference on the glycolipid metabolism of the fish. The digestive enzyme activities of low to medium CHO:L ratio were significantly increased after carbohydrase supplementation. Some studies revealed that the addition of cellulases to duckweed diet could significantly promote the protease, amylase and lipase activities of grass carp [68]; the exogenous xylanase supplementation in plant-protein-enriched diets reported on Jian carp (*Cyprinus carpio*) [69]; and a commercial enzyme complex of neutral protease, β-glucanase and xylanase supplementation in diets increased amylase and protease activities in tilapia [70]. Therefore, carbohydrase supplementation at an appropriate carbohydrate–lipid ratio can improve the digestive enzyme activity, improve the digestive ability and promote the growth performance of groupers. 

Once fish are exposed to the surrounding aquatic environment and live bait, a variety of bacteria begin to colonize the intestine, and the first colonized bacteria are adapted to the host intestinal environment, so the intestine is the largest number of bacteria in the digestive tract [71]. Studies on grass carp [68] have reported that cellulase supplementation promotes the emergence of certain flora, such as *Bacillus* and *Sphingomonas*, which aid in the digestion of cellulose. *Myroides* was related to severe skin and soft tissue infections, including amputation site and urinary tract infections, ventricular ventriculitis and bacteremia [72]. *Alistipes* is a producer of acetic acid, a short-chain fatty acid that has been shown to have anti-inflammatory mechanisms in previous studies, and it can be assumed that a decrease in *Alistipes* causes a decrease in short-chain fatty acids [73]. Therefore, to a certain extent, the supplementation of exogenous enzymes promotes the growth of beneficial flora and inhibits the reproduction of harmful flora. *Rikenellaceae* is able to use glucose from the environment to produce acetic acid [74], thus eliminating glucose from the surrounding environment. Studies based on 16S rRNA sequencing have found that among them, *Chryseobacterium*, *Cloacibacterium* and *Flavobacterium* belong to the anerobic class *Flavobacteriales*, which are known for their metabolism of lipids and carbohydrates [75]. This was a result consistent with the emergence of communities that contributed to the digestion of carbohydrates after supplementation with carbohydrase in our experiments.

In summary, our experimental results showed that the best growth performance and feed utilization were obtained at a dietary CHO:L ratio of 1.92; carbohydrase supplementation at dietary CHO:L ratio of 3.91 can significantly improve growth performance, digestive enzymes, glycolytic enzymes, *glut2* expression in the intestine, and the appearance of bacteria that contributed to the digestion of carbohydrates. Overall, it is a feasible strategy for carbohydrase supplementation with an appropriately high CHO: L ratio.

## Figures and Tables

**Table 1:** Formulation and proximate analysis of trial diets (dry matter, DM %).

Ingredients (%)	Groups					
	L	M	H	LE	ME	HE
Fishmeal ^a^	26	26	26	26	26	26
Casein ^a^	25	25	25	25	25	25
Wheat gluten ^a^	6	6	6	6	6	6
Corn starch ^a^	15	24	33	15	24	33
Fish oil ^a^	12.63	8.63	4.37	12.63	8.63	4.37
Soybean lecithin ^a^	1	1	1	1	1	1
Vitamin premix ^b^	0.2	0.2	0.2	0.2	0.2	0.2
Mineral premix ^c^	0.5	0.5	0.5	0.5	0.5	0.5
Enzyme	0	0	0	0.05	0.05	0.05
Calcium monophosphate ^d^	1	1	1	1	1	1
Choline chloride ^d^	0.5	0.5	0.5	0.5	0.5	0.5
Antioxidants ^a^	0.03	0.03	0.03	0.03	0.03	0.03
Attractant ^a^	0.1	0.1	0.1	0.1	0.1	0.1
Vitamin C ^a^	0.05	0.05	0.05	0.05	0.05	0.05
Carboxymethyl cellulose ^e^	2	2	2	2	2	2
Microcrystalline cellulose ^e^	9.99	4.99	0.25	9.94	4.94	0.25
Total	100.00	100.00	100.00	100.00	100.00	100.00
Proximate composition						
Moisture	10.85	10.06	11.11	9.57	10.62	10.67
Crude protein	43.20	43.14	44.12	43.42	43.49	43.57
Crude lipid	15.96	12.27	8.03	16.06	12.05	8.73
Ash	6.46	6.3	6.25	6.18	6.24	6.34
Carbohydrate	15	24	33	15	24	33
Carbohydrate/lipid	0.91	1.92	3.91	0.91	1.92	3.91
Gross energy (MJ Kg^−1^)	19.62	19.58	19.54	19.62	19.58	19.54

^a^ Ingredients were purchased from Zhanjiang, Hai Bao Factory, Zhanjiang, Guangdong, China. ^b^ Premix supplied the following vitamins (IU or mg/kg): Vitamin A, 900,000 IU; Vitamin D, 200,000 IU; Vitamin E, 4500 mg; Vitamin K3, 220 mg; Vitamin B1, 320 mg; Vitamin B2, 1090 mg; Niacin, 2800 mg; Vitamin B5, 2000 mg; Vitamin B6, 500 mg; Vitamin B12, 1.6 mg; Vitamin C, 5000 mg; Pantothenate, 1000 mg; Folic acid, 165 mg; Choline, 60,000 mg (Obtained from Zhanjiang Yue Hai Feed Co. Ltd., Guangdong China). ^c^ Premix suppled the following minerals (g/kg): CuSO_4_·5H_2_O, 2.0 g; FeSO_4_·7H2O, 25g; ZnSO_4_·7H_2_O, 22 g; MnSO_4_·4H_2_O, 7g; Na_2_SeO_3_, 0.04 g; KI, 0.026g; CoCl_2_·6H_2_O, 0.1 g (Obtained from Zhanjiang Yue Hai Feed Co., Ltd., Guangdong China). ^d^ Obtained from Shanghai Macklin Biochemical Co., Ltd., Shanghai China. ^e^ Acquired from Shantou Xilong Chemical Factory, Guangdong China.

**Table 2:** The primer sequences designed for the quantitative expression of *glut2* mRNA.

Primer name	Forward (5′-3′)	Reverse (5′-3′)	Accession No.
*Glut2*	CTCCGATTCCAGAACGACTACTC	TGGAATAGGACCAGGACCGAT	KY656467.1
*β-Actin*	TACGAGCTGCCTGACGGACA	GGCTGTGATCTCCTTCTGC	AY510710.2

N = A/C/G/T; D = A/G/T; B = C/G/T; S = C or G; Y = C or T; R = A or G; W = A or T; K = G or T.

**Table 3:** Effects of carbohydrase supplementation on growth performance, survival and feed utilization in hybrid grouper fed different CHO:L ratios diets (n = 3).

Groups	CHO:L	Enzyme	IBW(g)	FBW(g)	WGR(%)	SGR(% day^−1^)	SR(%)	FCR
L	0.91		9.78 ±0.01 ^a^	44.48 ± 4.39 ^a^	333.40 ± 26.71 ^a^	2.82 ± 0.12 ^a^	95.56 ± 3.85 ^a^	1.20 ± 0.08 ^b^
M	1.92		9.78 ± 0.00 ^a^	54.27 ± 2.38 ^b^	448.60 ± 22.61 ^cd^	3.27 ± 0.08 ^cd^	98.89 ± 1.92 ^a^	0.96 ± 0.10 ^a^
H	3.91		9.78 ± 0.01 ^a^	50.66 ± 2.55 ^ab^	412.05 ± 21.21 ^bc^	3.14 ± 0.08 ^bc^	98.89 ± 1.92 ^a^	1.07 ± 0.07 ^ab^
LE	0.91	E	9.78 ± 0.01 ^a^	47.12 ± 1.35 ^a^	371.00 ± 14.35 ^ab^	2.98 ± 0.06 ^ab^	97.78 ± 1.92 ^a^	1.16 ± 0.04 ^b^
ME	1.92	E	9.78 ± 0.01 ^a^	56.39 ± 2.21 ^b^	463.61 ± 19.12 ^cd^	3.32 ± 0.07 ^cd^	97.78 ± 1.92 ^a^	0.94 ± 0.08 ^a^
HE	3.91	E	9.78 ± 0.00 ^a^	57.02 ± 1.26 ^b^	476.41 ± 7.97 ^d^	3.36 ± 0.03 ^d^	98.89 ± 1.92 ^a^	0.93 ± 0.06 ^a^
Means of main effect								
L	0.91		9.78	45.80 ^A^	352.20 ^A^	2.90 ^A^	96.67	1.19 ^B^
M	1.92		9.78	55.33 ^B^	456.11 ^B^	3.30 ^B^	98.33	0.95 ^A^
H	3.91		9.78	53.84 ^B^	444.23 ^B^	3.25 ^B^	98.89	1.00 ^A^
		No	9.78	49.80 ^X^	398.02 ^X^	3.08 ^X^	97.78	1.082
		Have	9.78	53.51 ^Y^	437.01 ^Y^	3.22 ^Y^	98.15	1.012
Two-way ANOVA: *p*-values								
CHO:L			1.00	0.010	<0.001	<0.001	0.194	<0.001
Enzyme			0.78	<0.001	0.001	0.002	0.090	0.06
CHO:L*Enzyme			0.49	0.333	0.135	0.178	0.115	0.30

Weight gain rate (WGR, %) = 100 × (final body weight (g) − initial body weight (g))/initial bodyweight (g). Specific growth rate (SGR, %) = 100 × (ln final body weight (g) − ln initial body weight (g))/days of experiment. Feed conversion ratio (FCR) = feed intake (g)/(final body weight (g) − initial body weight(g)). Survival rate (SR, %) = 100 × (final fish number)/initial fish number. Value was expressed as the means of three replications (n = 3). Average of different superscripts in the same column (a, b, c, d etc. Or A, B etc. Or X, Y) are significantly different (*p* < 0.05). (Dietary CHO:L ratio= A, B etc.; Dietary carbohydrase = X, Y). L, M, and H represent CHO:L ratios of 0.91, 1.92 and 3.91, respectively. LE, ME, and HE represent CHO:L ratios of 0.91, 1.92, 3.91, respectively, supplemented with the same ratio of carbohydrase; “No” means no enzyme, “Have” and “E” means enzyme.

**Table 4:** Effects of carbohydrase supplementation on body composition in hybrid grouper fed different CHO:L ratios diets (n = 3).

Groups	CHO:L	Enzyme	Moisture	Crude Protein	Crude Lipid	Ash
L	0.91		66.70 ± 4.56	58.42 ± 2.09	28.50 ± 1.74 ^c^	14.53 ± 0.41
M	1.92		69.93 ± 1.43	58.36 ± 1.40	26.26 ± 2.19 ^bc^	13.90 ± 0.57
H	3.91		70.92 ± 9.17	59.06 ± 1.60	22.29 ± 2.28 ^ab^	13.85 ± 0.74
LE	0.91	E	74.02 ± 6.92	57.33 ± 2.54	28.27 ± 1.66 ^c^	13.68 ± 0.13
ME	1.92	E	67.83 ± 5.52	58.42 ± 1.52	27.37 ± 1.82 ^bc^	13.80 ± 0.36
HE	3.91	E	71.62 ± 3.47	59.59 ± 2.36	19.60 ± 1.79 ^a^	14.56 ± 0.52
Means of main effect						
L	0.91		70.36	57.87	28.39 ^B^	14.11
M	1.92		68.88	58.39	26.82 ^B^	13.85
H	3.91		71.27	59.33	20.94 ^A^	14.21
		No	69.18	58.611	25.69	14.09
		Have	71.16	58.447	25.08	14.01
Two-way ANOVA: *p*-values						
CHO:L			0.770	0.456	<0.001	0.453
Enzyme			0.479	0.862	0.521	0.740
CHO:L*Enzyme			0.373	0.767	0.262	0.055

Value was expressed as the means of three replications (n = 3). Average of different superscripts in the same column (a, b, c, etc. Or A, B etc. Or X, Y) are significantly different (*p* < 0.05). (Dietary CHO:L ratio = A, B etc.; Dietary carbohydrase = X, Y). L, M, and H represent CHO:L ratios of 0.91, 1.92 and 3.91, respectively. LE, ME, and HE represent CHO:L ratios of 0.91, 1.92, 3.91, respectively, supplemented with the same ratio of carbohydrase; “No” means no enzyme, “Have” and “E” means enzyme.

**Table 5:** Effects of carbohydrase supplementation on plasma biochemistry in hybrid grouper fed different CHO:L ratios diets.

Groups	CHO:L	Enzyme	TG (mmol/L)	TC (mmol/L)	GLU (mmol/mL)	INS (m U/L)
L	0.91		0.61 ± 0.04 ^d^	2.87 ± 0.08 ^d^	4.79 ± 0.09 ^b^	29.59 ± 0.70 ^a^
M	1.92		0.49 ± 0.04 ^c^	1.79 ± 0.21 ^c^	6.10 ± 0.38 ^c^	38.86 ± 0.49 ^b^
H	3.91		0.41 ± 0.06 ^abc^	1.14 ± 0.10 ^ab^	7.46 ± 0.17 ^d^	47.64 ± 2.25 ^cd^
LE	0.91	E	0.49 ± 0.02 ^bc^	1.90 ± 0.03 ^c^	4.24 ± 0.25 ^b^	44.37 ± 1.41 ^c^
ME	1.92	E	0.39 ± 0.02 ^ab^	1.40 ± 0.16 ^b^	4.18 ± 0.58 ^b^	50.96 ± 1.71 ^d^
HE	3.91	E	0.31 ± 0.04 ^a^	0.89 ± 0.20 ^a^	3.10 ± 0.25 ^a^	58.34 ± 1.29 ^e^
Means of main effect						
L	0.91		0.55 ^C^	2.39 ^C^	4.52 ^A^	36.98 ^A^
M	1.92		0.44 ^B^	1.60 ^B^	5.14 ^B^	44.91 ^B^
H	3.91		0.36 ^A^	1.02 ^A^	5.28 ^B^	52.99 ^C^
		No	0.50 ^X^	1.936 ^X^	6.11 ^X^	38.69 ^X^
		Have	0.40 ^Y^	1.396 ^Y^	3.84 ^Y^	51.22 ^Y^
Two-way ANOVA: *p*-values						
CHO:L			<0.001	<0.001	0.004	<0.001
Enzyme			<0.001	<0.001	<0.001	<0.001
CHO:L*Enzyme			0.791	0.002	<0.001	0.081

Abbreviations: TG: triglycerides; TC: cholesterol; GLU: glucose; INS: insulin. Value was expressed as the means of three replications (n = 3). Average of different superscripts in the same column (a, b, c, d, etc. Or A, B, C, etc. Or X, Y) are significantly different (*p* < 0.05). (Dietary CHO:L ratio = A, B, C, etc.; Dietary carbohydrase = X, Y). L, M, and H represent CHO:L ratios of 0.91, 1.92 and 3.91, respectively. LE, ME, and HE represent CHO:L ratios of 0.91, 1.92, 3.91, respectively, supplemented with the same ratio of carbohydrase; “No” means no enzyme, “Have” and “E” means enzyme.

**Table 6:** Effects of carbohydrase supplementation on intestinal digestive enzymes in hybrid grouper fed different CHO:L ratios diets.

Groups	CHO:L	Enzyme	Trypsin(U/mg Prot)	Amylase(IU/mg Prot)	Lipase(mU/mg Prot)
L	0.91		2868.34 ± 117.13 ^a^	252.22 ± 14.46 ^a^	1115.49 ± 23.44 ^b^
M	1.92		3997.42 ± 156.84 ^bc^	553.33 ± 12.79 ^b^	960.36 ± 26.88 ^a^
H	3.91		3933.64 ± 160.74 ^bc^	673.75 ± 8.98 ^c^	920.68 ± 36.56 ^a^
LE	0.91	E	3585.75 ± 166.99 ^b^	261.73 ± 27.35 ^a^	1131.23 ± 52.55 ^b^
ME	1.92	E	4457.23 ± 155.06 ^d^	618.85 ± 33.17 ^c^	1006.00 ± 19.95 ^a^
HE	3.91	E	4281.28 ± 178.43 ^cd^	1000.99 ± 22.07 ^d^	933.43 ± 27.09 ^a^
Means of main effect					
L	0.91		3227.04 ^A^	256.98 ^A^	1123.36 ^C^
M	1.92		4227.32 ^B^	586.09 ^B^	983.18 ^B^
H	3.91		4107.46 ^B^	837.36 ^C^	927.06 ^A^
		No	3599.80 ^X^	493.10 ^X^	998.84
		Have	4108.09 ^Y^	627.19 ^Y^	1023.55
Two-way ANOVA: *p*-values					
CHO:L			<0.001	<0.001	<0.001
Enzyme			<0.001	<0.001	0.137
CHO:L*Enzyme			0.155	<0.001	0.645

Value is expressed as the means of three replications (n = 3). Averages of different superscripts in the same column (a, b, c, d, etc. Or A, B, C, etc. Or X, Y) are significantly different (*p* < 0.05). (Dietary CHO:L ratio = A, B, C, etc.; Dietary carbohydrase = X, Y). L, M, H represent the different glycolipid ratios of 0.91, 1.92, 3.91, respectively. L, M, and H represent CHO:L ratios of 0.91, 1.92 and 3.91, respectively. LE, ME, and HE represent CHO:L ratios of 0.91, 1.92, 3.91, respectively, supplemented with the same ratio of carbohydrase; “No” means no enzyme, “Have” and “E” means enzyme.

## Data Availability

The data presented in this study are available in the main article.
